# Assessment Battery for Communication (ABaCo): An Extension of Normative Data to Elderly Individuals

**DOI:** 10.1007/s10936-026-10264-7

**Published:** 2026-06-01

**Authors:** Romina Angeleri, Ilaria Gabbatore, Ariele Merlini, Francesca M. Bosco

**Affiliations:** 1 Department of Theoretical and Applied Sciences, eCampus University, Novedrate (CO), Italy; 2 Department of Humanities, University of Turin, Turin, Italy; 3 Research Group on Inferential Processes in Social Interaction, GIPSI, University of Turin, Turin, Italy; 4 Department of Psychology, University of Turin, Turin, Italy; 5 Neuroscience Institute of Turin, University of Turin, Turin, Italy

**Keywords:** Normative data, Pragmatic assessment, Pragmatic disorders, Elderly population

## Abstract

The aim of this study is to update and expand the normative data for the Assessment Battery for Communication (ABaCo) to include older adults. As the population continues to age, comprehensive assessments of pragmatic ability become increasingly important, as deficits in this area can significantly impair social functioning. The ABaCo is a validated instrument designed to evaluate communicative-pragmatic ability in individuals with brain injuries, as well as neuropsychological and psychiatric disorders. Previously published normative data covered individuals aged 15 to 75 years. In this study, we present updated norms for that age range, based on an Italian enlarged sample (*N* = 409), and provide additional normative data for individuals aged 75 to 86 years (*N* = 33). Given that prior research has shown ABaCo performance declines with age and lower educational attainment, the normative data were stratified by both age and education level. Accurate identification of pragmatic-communicative impairments is essential in clinical settings, especially for the development of targeted interventions. These extended norms aim to improve the clinical utility and diagnostic accuracy of the ABaCo.

## Introduction

The Assessment Battery for Communication (ABaCo; Angeleri, 2012) is one of the few validated tools designed to evaluate communicative-pragmatic ability in individuals with brain lesions, as well as neuropsychological and psychiatric disorders. ABaCo was developed to enable the clinical assessment of the pragmatic aspects of communication, providing clinicians with a structured and reliable tool to evaluate how individuals use language and nonverbal cues effectively in social interactions. ABaCo demonstrated good psychometric properties and is available in two equivalent forms (Bosco et al., 2012), which include items differing in content but matched for complexity, allowing for test-retest procedures. Pragmatics refers to the use of language (Levinson, 1983) and other expressive means—such as non-verbal and extralinguistic cues (Bara, 2010; Holler & Levinson, 2019)—within context. It encompasses how meaning is constructed and interpreted beyond the literal meaning of words, depending on the situation, the speaker’s intentions, and the listener’s perspective. Given that pragmatic competence underpins the ability to produce contextually appropriate discourse and to navigate social interactions and relationships effectively (e.g., Adams, 2002; Milligan et al., 2007), impairments in this domain can have a substantial negative impact on individuals’ quality of life (Snow & Douglas, 2017). Deficits in pragmatic ability can severely hinder patients’ social functioning (Douglas, 2017; Struchen et al., 2008), affect the quality of their romantic relationships (Kreutzer et al., 2016; Van den Broek et al., 2022), reduce employment opportunities (Douglas et al., 2016), and ultimately diminish overall well-being (Snow & Douglas, 2017).

Pragmatic impairments have been documented across a range of neurological conditions, including traumatic brain injury (Angeleri et al. 2008; Bosco et al. 2017, 2018a; Büttner-Kunert et al. 2022), autism spectrum disorder (Angeleri et al., 2016; Simmons et al., 2014), vascular lesions (e.g., Gabbatore et al., 2014; Parola et al., 2016; Silagi et al., 2018; Spaccavento et al., 2024), Parkinson’s disease (Baraldi et al., 2021; Montemurro et al., 2019), and frontotemporal dementia (Cummings, 2020; Spotorno et al., 2015). Similar deficits have also been reported in psychiatric conditions such as schizophrenia (Agostoni et al., 2024; Bosco et al., 2019; Morese et al., 2022; Parola et al., 2020). Moreover, evidence of communicative-pragmatic decline has emerged in the context of healthy aging (Baraldi and Domaneschi 2024; Bambini et al. 2021; Hilviu et al. 2022, 2025; Gabbatore et al. 2025a, 2025c).

ABaCo is grounded in a robust and coherent theoretical framework, namely the Cognitive Pragmatics Theory (Bara, 2010), and assesses communicative competence across five distinct domains: linguistic, extralinguistic, paralinguistic, contextual, and conversational ability. This instrument enables the detailed identification of pragmatic impairments, offering a thorough profile of a patient’s strengths and weaknesses to inform tailored rehabilitation strategies. Moreover, an adapted version of the ABaCo is available, specifically designed to assess pragmatic ability across different developmental populations (Angeleri et al., 2016; Bosco et al., 2013). ABaCo has proven effective in detecting pragmatic impairments across a variety of clinical populations, including individuals with traumatic brain injury (Angeleri et al., 2008), left- and right-hemisphere brain lesions (Gabbatore et al., 2014; Parola et al., 2016), schizophrenia (Colle et al., 2013), adolescents in the autism spectrum (Angeleri et al., 2016; Gabbatore et al., 2022), children with cochlear implants (Parola et al., 2023), and those with Social Communication Pragmatic Disorder (Gabbatore et al., 2025b). Furthermore, ABaCo has been partially adapted to several cultural contexts (Agrela et al., 2020; Davis et al., 2016; Đorđević et al., 2016; Gabbatore et al., 2019). More recently, an online version of the tool (e-ABaCo) has been developed, enabling its use in tele-practice settings (Traetta et al., 2025).

In order for ABaCo to be effectively utilized in clinical settings, we provided normative data that serve as a reference point for interpreting patients’ performance, taking into account the influence of socio-demographic variables such as age and education (Angeleri et al., 2012). The objective of the present study is twofold: first, to revise and extend previously published normative data; and second, to broaden the age range covered by these normative standards, including participants up to 86 years of age. Extending the normative sample to include older individuals is particularly important in light of the steady increase in life expectancy worldwide (World Health Organization, 2024), especially in Italy, where ABaCo was developed and where this demographic trend is particularly pronounced (Italian Institute for International Political Studies, 2023). Monitoring communication decline associated with aging is becoming increasingly important, particularly given that pragmatic assessment has been recognized as a reliable indicator of cognitive impairment (Lago et al., 2022; Sherman et al., 2021), and that advancing age represents the primary risk factor for neurodegenerative diseases and neuropsychological disorders (Hou et al., 2019).

## Method

### Participants

To update and expand the ABaCo normative data, 141 participants were recruited for the present study via classroom announcements and convenience sampling through the research group’s extensive network. These data were added to the existing normative dataset, resulting in a total sample of 442 participants. All participants were native Italian speakers residing in Italy, within the target age range (15–86 years), and with available information on age and education. Participants were included if they met these criteria and had no history or current presence of psychiatric or neurological disorders, no significant medical conditions within the year preceding participation (e.g., cancer, coronary artery disease), no use of psychotropic drugs, and no history or current use of alcohol or drug abuse. All participants provided informed consent prior to participation. The sample was stratified based on age and years of education, following the guidelines provided by the Italian National Institute of Statistics (ISTAT), to ensure it represents the overall national population. The final sample included 442 individuals, organized as follows: 409 individuals aged between 15 and 74 years (*M*: 45.09, *SD*: 16.99, 48.2% female), and 33 individuals aged between 75 and 86 years (*M*: 80.64, *SD*: 3.53; 54.5% female), with the goal of extending the normative data to older age groups. Table 1 provides an overview of the distribution of individuals across each age and education groups.

**Table 1:** Distribution of participants by age and education group

Education^a^ (years of schooling)	Age group				
	15–34	35–54	55–74	75–86	Total
5	–	5	52	10	68
8	52	61	34	10	156
13	63	47	32	7	149
17+	25	18	20	6	69
Total	140	131	138	33	442

a Education was stratified according to the Italian education system, which includes primary school (5 years, starting at age 6 and mandatory), middle school (3 years, starting at age 11 and mandatory), high school (5 years, starting at age 14 and mandatory until age 16), and university (5 to 6 years, depending on the field of study)

### Materials

#### ABaCo (Assessment Battery for Communication)

The Assessment Battery for Communication (ABaCo) is a clinical instrument designed to evaluate multiple components of communicative-pragmatic competence across different modalities. The full battery comprises 180 items, including 72 examiner-delivered prompts and 108 brief videotaped scenes (each lasting 20–25 s), and requires approximately 90 min to administer. Each scale can also be administered independently for clinical or research purposes. The battery consists of five scales: Linguistic, Extralinguistic, Paralinguistic, Context, and Conversational.

#### Linguistic Scale

The scale assesses both the comprehension and production of communicative acts linguistically expressed. It encompasses basic communicative acts (assertions, questions, requests, and commands, see Kasher, 1981), standard acts (simple and complex), and non-standard acts (simple and complex forms of irony and deceit). Comprehension tasks require participants to interpret linguistically expressed communicative acts presented either directly by the examiner or within brief video-based interactions between two interlocutors. Production tasks require participants to generate appropriate verbal responses, either by producing specific basic acts based on provided semantic content or by responding to a communicative exchange depicted in a video.

#### Extralinguistic Scale

The scale evaluates both the comprehension and production of communicative acts conveyed exclusively through gestures. It mirrors the structure and categories of the Linguistic scale (basic, standard, irony and deceit). In comprehension tasks, participants interpret gestural communicative acts presented in brief video clips. In production tasks, they generate appropriate gestural responses, either by following the examiner’s instructions or by responding to a gestural interaction depicted in a video.

#### Paralinguistic Scale

This scale examines both the comprehension and production of communicative information conveyed through prosody, facial expression, and other paralinguistic cues accompanying speech. It includes (a) basic communicative acts, (b) acts expressing basic emotions (e.g., happiness, sadness, anger, fear), and (c) acts characterized by paralinguistic contradiction, in which verbal content conflicts with paralinguistic signals (assessed in comprehension only). To isolate paralinguistic processing, comprehension items feature videos in which actors speak an invented language, requiring participants to rely exclusively on prosodic and expressive cues. In production tasks, participants are required to modulate prosody and facial expression appropriately, either to produce neutral communicative acts or to convey a specified emotional tone.

#### Context Scale

The scale assesses sensitivity to discourse and social norms. Comprehension tasks require participants to detect and explain violations of discourse principles (e.g., lack of informativeness, irrelevance, and ambiguity, see Brown & Yule, 1983; Grice, 1975) or breaches of social appropriateness within video-based interactions. Production tasks require participants to generate communicative acts that are suitable for a given social context, modulating formality and politeness according to situational demands.

#### Conversational Scale

This scale evaluates participants’ ability to engage effectively in a semi-structured conversation with the examiner (lasting approximately 4–6 min). Two topics are introduced, and participants are assessed on their capacity to maintain topic coherence, adhere to turn-taking rules, and contribute responses that are both relevant and contextually appropriate. In this scale, comprehension and production processes are inherently integrated within the interactive exchange.

### Procedure

Elderly participants enrolled in the study were screened based on their self-reported cognitive and health changes over the past year. Those who met the inclusion criteria were invited to participate in the study, and a specific day and time were arranged for data collection. The interviews were conducted in the participants’ homes with a research assistant specifically trained to rigorously apply the ABaCo procedures; each participant completed two sessions, each lasting approximately one hour. The participants viewed the stimuli on a laptop, and the in-person items were conducted by the research assistant. The experimental sessions were video recorded in their entirety, allowing the recordings to be reviewed later for accurate coding and scoring of participants’ responses. Consent was obtained from all participants prior to their participation, and the study was approved by the Bioethical Committee of the University of Turin (Prot. No. 0193556).

### Scoring Procedure

Two independent raters coded the participants’ responses without knowing the objectives of the study. They filled in their scores on specific score sheets while viewing the participants’ video-recorded experimental sessions. Each answer could receive a score of either 0 or 1: a score of 1 was given for correct answers, and 0 for incorrect ones (for a detailed explanation of the scoring criteria (see Angeleri et al., 2008; Sacco et al., 2008).

## Results

### Tabular Norms

Normative data for various combinations of age and education are shown in Table 2. For each subgroup, the table shows the mean, standard deviation (SD), median, minimum/maximum values, skewness, and kurtosis of scores on the ABaCo scales and subscales, plus the composite scores for comprehension and production and global ABaCo score.

**Table 2:** Statistical properties for age, education, and ABaCo scales for each normative group

Age groups			Statistics				
			Mean (SD)	Median	Minimum-maximum	Skewness	Kurtosis
*Age group 15–34 (N = 140)*							
*Education 8 years (N = 52)*							
Age			24.3 (5.82)	24.00	16–34		
Global score (all tasks)			0.91 (0.05)	0.91	0.76 − 0.98	− 0.80	0.48
Comprehension (all tasks)			0.88 (0.06)	0.88	0.72 − 1.00	− 0.37	− 0.05
Production (all tasks)			0.92 (0.07)	0.94	0.65 − 1.00	−1.76	4.29
Linguistic scale			0.92 (0.06)	0.93	0.72 − 1.00	− 0.86	0.39
Linguistic comprehension			0.94 (0.07)	0.96	0.70 − 1.00	−1.07	0.57
Linguistic production			0.90 (0.08)	0.91	0.71 − 1.00	− 0.81	− 0.2
Extralinguistic scale			0.89 (0.07)	0.90	0.63 − 0.98	−1.23	2.62
Extralinguistic comprehension			0.89 (0.11)	0.89	0.73 − 1.00	− 0.12	− 0.26
Extralinguistic production			0.88 (0.2)	0.89	0.33 − 1.00	−2.64	11.7
Paralinguistic scale			0.90 (0.08)	0.92	0.55 − 1.00	−1.82	5.45
Paralinguistic comprehension			0.87 (0.08)	0.88	0.63 − 1.00	− 0.55	0.28
Paralinguistic production			0.93 (0.13)	0.96	0.25 − 1.00	−3.87	17.7
Context scale			0.88 (0.06)	0.91	0.61 − 1.00	−1.49	3.39
Context comprehension			0.85 (0.13)	0.86	0.35 − 1.00	−1.28	2.79
Context production			0.97 (0.07)	1.00	0.75 − 1.00	−2.54	5.53
Conversational scale			0.95 (0.08)	1.00	0.61 − 1.00	−1.98	5.79
*Age group 15–34 (N = 140)*							
*Education 13 years (N = 63)*							
Age			27.7 (3.71)	28.00	20–34		
Global score (all tasks)			0.92 (0.04)	0.93	0.80 − 0.99	− 0.89	0.52
Comprehension (all tasks)			0.91 (0.05)	0.91	0.78 − 1.00	− 0.77	0.54
Production (all tasks)			0.92 (0.06)	0.94	0.73 − 1.00	−1.23	1.28
Linguistic scale			0.94 (0.05)	0.95	0.75 − 1.00	−1.21	1.63
Linguistic comprehension			0.96 (0.05)	0.96	0.79 − 1.00	−1.32	1.34
Linguistic production			0.92 (0.07)	0.93	0.71 − 1.00	− 0.86	0.16
Extralinguistic scale			0.89 (0.09)	0.90	0.60 − 1.00	−1.38	2.11
Extralinguistic comprehension			0.93 (0.06)	0.93	0.75 − 1.00	− 0.69	0.27
Extralinguistic production			0.84 (0.15)	0.89	0.36 − 1.00	−1.46	2.37
Paralinguistic scale			0.91 (0.06)	0.92	0.74 − 1.00	− 0.62	0.14
Paralinguistic comprehension			0.86 (0.07)	0.85	0.70 − 1.00	− 0.36	0.02
Paralinguistic production			0.95 (0.08)	1.00	0.58 − 1.00	−2.07	5.47
Context scale			0.93 (0.06)	0.94	− 0.75 − 1.00	−1.00	0.32
Context comprehension			0.87 (0.12)	0.93	0.5 − 1.00	−1.15	0.94
Context production			0.98 (0.06)	1.00	0.75 − 1.00	−3.1	8.86
Conversational scale			0.97 (0.05)	1.00	0.79 − 1.00	−1.74	1.84
*Age group 15–34 (N = 140)*							
*Education 17 years (N = 25)*							
Age			26.7 (3.23)	26.00	22–33		
Global score (all tasks)			0.95 (0.03)	0.96	0.89 − 1.00	− 0.51	− 0.55
Comprehension (all tasks)			0.94 (0.03)	0.94	0.87 − 1.00	− 0.05	− 0.43
Production (all tasks)			0.96 (0.05)	0.97	0.84 − 1.00	−1.17	0.78
Linguistic scale			0.97 (0.04)	0.98	0.89 − 1.00	−1.12	0.13
Linguistic comprehension			0.98 (0.03)	1.00	0.93 − 1.00	− 0.72	− 0.98
Linguistic production			0.94 (0.09)	0.96	0.64 − 1.00	−1.94	4.38
Extralinguistic scale			0.94 (0.05)	0.95	0.85 − 1.00	− 0.70	− 0.74
Extralinguistic comprehension			0.93 (0.05)	0.95	0.86 − 1.00	− 0.40	−1.06
Extralinguistic production			0.92 (0.09)	0.96	0.68 − 1.00	−1.18	0.83
Paralinguistic scale			0.93 (0.06)	0.94	0.77 − 1.00	−1.24	2.36
Paralinguistic comprehension			0.91 (0.07)	0.90	0.70 − 1.00	− 0.86	1.44
Paralinguistic production			0.95 (0.12)	1.00	0.58 − 1.00	−2.74	6.88
Context scale			0.94 (0.03)	0.96	0.85 − 1.00	−1.04	0.67
Context comprehension			0.94 (0.05)	0.93	0.82 − 1.00	− 0.56	− 0.33
Context production			0.98 (0.05)	1.00	0.88 − 1.00	−1.98	2.06
Conversational scale			0.97 (0.05)	1.00	0.86 − 1.00	−1.26	0.03
*Age group 35–54 (N = 131)*							
*Education 5 years (N = 5)*							
Age			47.4 (7.30)	52.00	38–53		
Global score (all tasks)			0.90 (0.04)	0.91	0.83 − 0.93	−1.4	1.71
Comprehension (all tasks)			0.86 (0.07)	0.89	0.73 − 0.90	−2.22	4.93
Production (all tasks)			0.91 (0.05)	0.92	0.85 − 0.96	− 0.28	− 0.55
Linguistic scale			0.91 (0.07)	0.93	0.79 − 0.95	−1.93	3.84
Linguistic comprehension			0.94 (0.08)	0.97	0.80 − 1.00	−1.67	2.81
Linguistic production			0.87 (0.07)	0.86	0.79 − 0.96	0.24	−1.96
Extralinguistic scale			0.88 (0.06)	0.89	0.77 − 0.94	−1.34	1.93
Extralinguistic comprehension			0.87 (0.12)	0.89	0.68 − 0.97	−1.78	3.64
Extralinguistic production			0.88 (0.04)	0.87	0.83 − 0.93	0.67	− 0.45
Paralinguistic scale			0.86 (0.05)	0.88	0.79 − 0.92	− 0.54	−1.31
Paralinguistic comprehension			0.79 (0.07)	0.79	0.70 − 0.88	− 0.17	− 0.99
Paralinguistic production			0.93 (0.08)	0.96	0.79 − 1.00	−1.94	4.17
Context scale			0.86 (0.07)	0.89	0.79 − 0.96	− 0.68	−1.04
Context comprehension			0.83 (0.08)	0.82	0.71 − 0.93	− 0.75	0.98
Context production			0.95 (0.07)	1.00	0.87 − 1.00	− 0.61	−3.33
Conversational scale			1.00 (0.00)	1.00	1.00–1.00	NaN	NaN
*Age group 35–54 (N = 131)*							
*Education 8 years (N = 52)*							
Age			44.2 (6.32)	44.00	35–54		
Global score (all tasks)			0.90 (0.05)	0.91	0.75 − 0.97	− 0.97	1.30
Comprehension (all tasks)			0.87 (0.06)	0.88	0.64 − 0.98	−1.19	2.22
Production (all tasks)			0.91 (0.06)	0.92	0.69 − 0.99	−1.21	2.39
Linguistic scale			0.90 (0.07)	0.91	0.71 − 1.00	− 0.98	0.78
Linguistic comprehension			0.94 (0.07)	0.96	0.70 − 1.00	−1.48	2.31
Linguistic production			0.87 (0.09)	0.86	0.50 − 1.00	−1.31	3.38
Extralinguistic scale			0.88 (0.09)	0.90	0.54 − 0.98	−1.61	3.28
Extralinguistic comprehension			0.88 (0.09)	0.90	0.45 − 1.00	−2.07	8.26
Extralinguistic production			0.87 (0.12)	0.90	0.52 − 1.00	−1.17	0.86
Paralinguistic scale			0.89 (0.05)	0.90	0.75 − 1.00	− 0.34	− 0.15
Paralinguistic comprehension			0.83 (0.09)	0.83	0.55 − 1.00	− 0.68	0.70
Paralinguistic production			0.95 (0.06)	0.96	0.67 − 1.00	−1.99	5.31
Context scale			0.91 (0.06)	0.94	0.49 − 1.00	−2.58	10.2
Context comprehension			0.85 (0.13)	0.88	0.24 − 1.00	−2.03	6.69
Context production			0.97 (0.06)	1.00	0.75 − 1.00	−1.83	2.6
Conversational scale			0.93 (0.08)	0.96	0.57 − 1.00	−1.85	6.23
*Age group 35–54 (N = 131)*							
*Education 13 years (N = 47)*							
Age			44.1 (6.11)	44.00	35–54		
Global score (all tasks)			0.92 (0.04)	0.92	0.83 − 0.98	− 0.64	− 0.39
Comprehension (all tasks)			0.88 (0.06)	0.89	0.74 − 0.98	− 0.72	− 0.05
Production (all tasks)			0.93 (0.04)	0.94	0.83 − 1.00	− 0.75	0.03
Linguistic scale			0.92 (0.05)	0.93	0.79 − 1.00	− 0.60	0.13
Linguistic comprehension			0.94 (0.06)	0.97	0.77 − 1.00	−1.26	0.73
Linguistic production			0.9 (0.08)	0.93	0.64 − 1.00	−1.14	1.25
Extralinguistic scale			0.90 (0.07)	0.91	0.65 − 1.00	−1.30	1.80
Extralinguistic comprehension			0.89 (0.08)	0.90	0.71 − 1.00	− 0.68	− 0.41
Extralinguistic production			0.91 (0.09)	0.93	0.59 − 1.00	−1.41	2.05
Paralinguistic scale			0.92 (0.06)	0.94	0.80 − 1.00	− 0.50	− 0.65
Paralinguistic comprehension			0.87 (0.09)	0.88	0.6 − 1.00	− 0.72	0.27
Paralinguistic production			0.97 (0.05)	1.00	0.83 − 1.00	−1.43	1.06
Context scale			0.90 (0.07)	0.91	0.71 − 1.00	− 0.69	0.14
Context comprehension			0.84 (0.13)	0.86	0.41 − 1.00	−1.21	2.17
Context production			0.96 (0.08)	1.00	0.75 − 1.00	−1.7	1.72
Conversational scale			0.97 (0.05)	1.00	0.83 − 1.00	−1.5	1.16
*Age group 35–54 (N = 131)*							
*Education 17 years (N = 18)*							
Age			43.8 (7.77)	43.5	35–54		
Global score (all tasks)			0.92 (0.03)	0.92	0.86 − 0.97	− 0.60	− 0.15
Comprehension (all tasks)			0.90 (0.04)	0.91	0.81 − 0.97	− 0.67	1.06
Production (all tasks)			0.93 (0.05)	0.91	0.82 − 0.99	− 0.31	− 0.28
Linguistic scale			0.93 (0.04)	0.93	0.80 − 0.98	−1.67	4.21
Linguistic comprehension			0.96 (0.04)	0.95	0.89 − 1.00	− 0.01	−1.06
Linguistic production			0.90 (0.07)	0.93	0.71 − 1.00	−1.2	2.16
Extralinguistic scale			0.91 (0.06)	0.92	0.79 − 0.99	− 0.48	−1.11
Extralinguistic comprehension			0.92 (0.05)	0.92	0.79 − 1.00	− 0.83	1.53
Extralinguistic production			0.89 (0.12)	0.91	0.67 − 1.00	− 0.85	− 0.44
Paralinguistic scale			0.92 (0.05)	0.92	0.83 − 0.98	− 0.59	− 0.44
Paralinguistic comprehension			0.88 (0.06)	0.88	0.75 − 0.96	− 0.13	− 0.51
Paralinguistic production			0.95 (0.06)	0.98	0.83 − 1.00	−1.06	− 0.05
Context scale			0.91 (0.07)	0.92	0.76 − 1.00	− 0.96	0.10
Context comprehension			0.87 (0.12)	0.87	0.53 − 1.00	−1.64	4.74
Context production			0.96 (0.09)	1.00	0.75 − 1.00	−1.91	2.44
Conversational scale			0.95 (0.08)	1.00	0.68 − 1.00	−2.48	6.95
*Age group 55–74 (N = 138)*							
*Education 5 years (N = 52)*							
Age			66.3 (5.35)	67.00	55–73		
Global score (all tasks)			0.87 (0.06)	0.88	0.72 − 0.95	− 0.99	0.77
Comprehension (all tasks)			0.83 (0.07)	0.83	0.69 − 0.95	− 0.05	− 0.91
Production (all tasks)			0.88 (0.08)	0.90	0.65 − 0.98	−1.31	1.50
Linguistic scale			0.89 (0.07)	0.90	0.67 − 0.98	− 0.89	0.90
Linguistic comprehension			0.92 (0.07)	0.93	0.73 − 1.00	− 0.94	− 0.05
Linguistic production			0.86 (0.09)	0.86	0.61 − 1.00	− 0.81	0.62
Extralinguistic scale			0.82 (0.08)	0.84	0.62 − 0.97	− 0.72	0.25
Extralinguistic comprehension			0.82 (0.08)	0.82	0.61 − 1.00	− 0.32	0.13
Extralinguistic production			0.82 (0.12)	0.83	0.52 − 1.00	− 0.67	− 0.24
Paralinguistic scale			0.84 (0.09)	0.85	0.51 − 0.98	−1.15	2.47
Paralinguistic comprehension			0.80 (0.11)	0.79	0.50 − 0.96	− 0.38	− 0.26
Paralinguistic production			0.92 (0.12)	0.96	0.42 − 1.00	−2.26	5.99
Context scale			0.87 (0.09)	0.88	0.64 − 1.00	− 0.62	− 0.50
Context comprehension			0.8 (0.14)	0.82	0.43 − 1.00	− 0.69	− 0.13
Context production			0.94 (0.1)	1.00	0.63 − 1.00	−1.48	1.04
Conversational scale			0.97 (0.06)	1.00	0.79 − 1.00	−2.03	2.95
*Age group 55–74 (N = 138)*							
*Education 8 years (N = 34)*							
Age			65.2 (5.78)	65.5	55–74		
Global score (all tasks)			0.87 (0.05)	0.88	0.75 − 0.96	− 0.65	− 0.02
Comprehension (all tasks)			0.85 (0.07)	0.84	0.68 − 0.99	− 0.13	0.28
Production (all tasks)			0.87 (0.09)	0.88	0.66 − 0.98	− 0.91	0.00
Linguistic scale			0.90 (0.07)	0.89	0.74 − 1.00	− 0.37	− 0.62
Linguistic comprehension			0.95 (0.05)	0.96	0.8 − 1.00	− 0.95	0.38
Linguistic production			0.85 (0.11)	0.86	0.64 − 1.00	− 0.43	− 0.84
Extralinguistic scale			0.85 (0.08)	0.87	0.63 − 0.97	− 0.84	0.09
Extralinguistic comprehension			0.86 (0.07)	0.89	0.66 − 0.97	− 0.66	− 0.26
Extralinguistic production			0.84 (0.14)	0.87	0.42 − 1.00	−1.15	1.39
Paralinguistic scale			0.83 (0.09)	0.85	0.58 − 0.95	−1.13	1.22
Paralinguistic comprehension			0.77 (0.11)	0.77	0.54 − 1.00	0.03	0.14
Paralinguistic production			0.90 (0.13)	0.94	0.50 − 1.00	−1.43	1.81
Context scale			0.86 (0.01)	0.88	0.54 − 1.00	−1.44	2.49
Context comprehension			0.82 (0.15)	0.84	0.33 − 1.00	−1.44	2.54
Context production			0.91 (0.14)	1.00	0.5 − 1.00	−1.46	1.23
Conversational scale			0.97 (0.05)	1.00	0.83 − 1.00	−1.61	1.21
*Age group 55–74 (N = 138)*							
*Education 13 years (N = 32)*							
Age			65.2 (5.63)	65.5	55–74		
Global score (all tasks)			0.88 (0.07)	0.89	0.72 − 0.98	− 0.73	− 0.34
Comprehension (all tasks)			0.85 (0.08)	0.84	0.67 (0.97)	− 0.39	− 0.68
Production (all tasks)			0.88 (0.1)	0.93	0.56 − 1.00	−1.20	0.64
Linguistic scale			0.91 (0.07)	0.91	0.75 − 1.00	− 0.64	− 0.44
Linguistic comprehension			0.93 (0.06)	0.93	0.82 − 1.00	− 0.67	− 0.57
Linguistic production			0.88 (0.1)	0.91	0.61 − 1.00	− 0.89	0.28
Extralinguistic scale			0.85 (0.1)	0.85	0.64 − 0.99	− 0.48	− 0.82
Extralinguistic comprehension			0.87 (0.1)	0.89	0.57 − 1.00	−1.25	1.93
Extralinguistic production			0.85 (0.14)	0.90	0.50 − 1.00	− 0.88	− 0.06
Paralinguistic scale			0.84 (0.1)	0.85	0.64 − 1.00	− 0.60	− 0.51
Paralinguistic comprehension			0.79 (0.13)	0.80	0.38 − 1.00	−1.22	2.52
Paralinguistic production			0.89 (0.16)	0.96	0.42 − 1.00	−1.71	1.93
Context scale			0.85 (0.1)	0.84	0.48 − 1.00	−1.41	1.85
Context comprehension			0.81 (0.16)	0.88	0.47 − 1.00	− 0.90	− 0.18
Context production			0.90 (0.19)	1.00	0.25 − 1.00	−2.75	7.72
Conversational scale			0.99 (0.03)	1.00	0.89 − 1.00	−2.54	5.71
*Age group 55–74 (N = 138)*							
*Education 17 years (N = 20)*							
Age			61 (6.44)	58.00	55–72		
Global score (all tasks)			0.88 (0.04)	0.89	0.78 − 0.95	− 0.80	0.52
Comprehension (all tasks)			0.87 (0.05)	0.87	0.76 − 0.94	− 0.86	0.10
Production (all tasks)			0.88 (0.08)	0.90	0.63 − 0.97	−1.60	3.31
Linguistic scale			0.89 (0.05)	0.89	0.81 − 1.00	0.19	− 0.05
Linguistic comprehension			0.93 (0.5)	0.93	0.83 − 1.00	− 0.51	− 0.21
Linguistic production			0.86 (0.07)	0.84	0.75 − 1.00	0.55	− 0.76
Extralinguistic scale			0.83 (0.07)	0.84	0.71 − 0.97	0.00	− 0.48
Extralinguistic comprehension			0.86 (0.06)	0.85	0.79 − 1.00	0.84	0.06
Extralinguistic production			0.78 (0.13)	0.82	0.57 − 0.93	− 0.37	−1.37
Paralinguistic scale			0.88 (0.07)	0.88	0.71 − 0.96	− 0.92	0.73
Paralinguistic comprehension			0.82 (0.08)	0.83	0.70 − 0.95	− 0.12	−1.12
Paralinguistic production			0.91 (0.13)	0.96	0.58 − 1.00	−1.53	0.96
Context scale			0.88 (0.12)	0.91	0.55 − 1.00	−1.50	2.24
Context comprehension			0.83 (0.13)	0.87	0.57 − 1.00	− 0.63	− 0.51
Context production			0.90 (0.18)	1.00	0.25 − 1.00	−2.76	9.06
Conversational scale			0.97 (0.04)	1.00	0.89 − 1.00	−1.04	− 0.66
*Age group 75–86 (N = 33)*							
*Education 5 years (N = 10)*							
Age			80.4 (3.63)	81.5	75–85		
Global score (all tasks)			0.76 (0.07)	0.77	0.65 − 0.85	− 0.16	−2.05
Comprehension (all tasks)			0.78 (0.07)	0.79	0.60 − 0.85	−1.81	4.39
Production (all tasks)			0.69 (0.12)	0.73	0.46 − 0.85	− 0.47	−1.35
Linguistic scale			0.81 (0.07)	0.83	0.64 − 0.88	−1.62	3.11
Linguistic comprehension			0.92 (0.13)	0.93	0.82 − 1.00	− 0.35	−1.09
Linguistic production			0.70 (0.13)	0.71	0.36 − 0.82	−2.47	7.22
Extralinguistic scale			0.64 (0.15)	0.65	0.38 − 0.82	− 0.46	− 0.71
Extralinguistic comprehension			0.73 (0.1)	0.73	0.57 − 0.86	− 0.45	−1.07
Extralinguistic production			0.56 (0.22)	0.54	0.11 − 0.82	− 0.67	0.08
Paralinguistic scale			0.68 (0.16)	0.71	0.43 − 0.87	− 0.45	−1.33
Paralinguistic comprehension			0.72 (0.14)	0.75	0.40 − 0.90	−1.22	1.6
Paralinguistic production			0.65 (0.24)	0.67	0.25 − 0.92	− 0.53	− 0.87
Context scale			0.80 (0.07)	0.76	0.70 − 0.93	0.49	−1.78
Context comprehension			0.74 (0.17)	0.79	0.43 − 0.93	− 0.6	− 0.67
Context production			0.85 (0.18)	0.88	0.50 − 1.00	− 0.78	− 0.15
Conversational scale			0.97 (0.07)	1.00	0.82 − 1.00	− 0.15	1.42
*Age group 75–86 (N = 33)*							
*Education 8 years (N = 10)*							
Age			80.4 (4.30)	81.00	75–86		
Global score (all tasks)			0.84 (0.09)	0.84	0.72 − 0.96	0.06	−1.72
Comprehension (all tasks)			0.83 (0.08)	0.83	0.71 − 0.97	0.38	− 0.33
Production (all tasks)			0.81 (0.14)	0.81	0.63 − 0.97	− 0.06	−1.88
Linguistic scale			0.86 (0.09)	0.88	0.66 − 0.96	−1.25	1.89
Linguistic comprehension			0.90 (0.08)	0.89	0.75 − 1.00	− 0.41	− 0.50
Linguistic production			0.83 (0.11)	0.87	0.57 − 0.93	−1.38	1.86
Extralinguistic scale			0.82 (0.12)	0.84	0.59 − 0.95	− 0.80	0.12
Extralinguistic comprehension			0.86 (0.09)	0.89	0.68 − 1.00	− 0.93	1.24
Extralinguistic production			0.77 (0.17)	0.79	0.50 − 0.96	− 0.61	− 0.80
Paralinguistic scale			0.76 (0.16)	0.76	0.47 − 1.00	− 0.20	− 0.30
Paralinguistic comprehension			0.77 (0.15)	0.78	0.55 − 1.00	0.35	− 0.28
Paralinguistic production			0.75 (0.24)	0.79	0.33 − 1.00	− 0.51	−1.13
Context scale			0.83 (0.11)	0.83	0.60 − 0.96	− 0.79	0.80
Context comprehension			0.79 (0.11)	0.79	0.57 − 0.93	− 0.62	0.29
Context production			0.88 (0.18)	1.00	0.50 − 1.00	−1.18	0.57
Conversational scale			0.98 (0.05)	1.00	0.85 − 1.00	−2.86	8.36
*Age group 75–86 (N = 33)*							
*Education 13 years (N = 7)*							
Age			81.7 (3.20)	80.00	78–86		
Global score (all tasks)			0.81 (0.06)	0.82	0.74 − 0.92	0.66	0.64
Comprehension (all tasks)			0.78 (0.08)	0.77	0.68 − 0.92	0.45	− 0.54
Production (all tasks)			0.81 (0.11)	0.80	0.60 − 0.95	− 0.81	1.35
Linguistic scale			0.79 (0.07)	0.81	0.65 − 0.88	−1.04	1.06
Linguistic comprehension			0.85 (0.08)	0.85	0.71 − 0.96	− 0.29	1.53
Linguistic production			0.72 (0.14)	0.75	0.48 − 0.93	− 0.29	0.47
Extralinguistic scale			0.80 (0.07)	0.79	0.68 − 0.87	− 0.51	− 0.46
Extralinguistic comprehension			0.79 (0.11)	0.82	0.61 − 0.92	− 0.64	− 0.17
Extralinguistic production			0.78 (0.11)	0.82	0.58 − 0.89	−1.17	0.95
Paralinguistic scale			0.76 (0.11)	0.78	0.56 − 0.93	− 0.56	0.86
Paralinguistic comprehension			0.70 (0.11)	0.70	0.60 − 0.85	1.18	3.00
Paralinguistic production			0.80 (0.22)	0.83	0.42 − 1.00	−1.03	− 0.05
Context scale			0.83 (0.1)	0.82	0.66 − 1.00	0.06	0.57
Context comprehension			0.76 (0.15)	0.71	0.57 − 1.00	0.50	− 0.97
Context production			0.93 (0.12)	1.00	0.75 − 1.00	−1.23	− 0.84
Conversational scale			0.98 (0.04)	1.00	0.89 − 1.00	−2.16	4.58
*Age group 75–86 (N = 33)*							
*Education 17 years (N = 6)*							
Age			80.2 (2.86)	80.5	76–84		
Global score (all tasks)			0.80 (0.09)	0.82	0.65 − 0.88	−1.11	0.82
Comprehension (all tasks)			0.84 (0.07)	0.86	0.73 − 0.90	−1.64	2.88
Production (all tasks)			0.72 (0.14)	0.73	0.50 − 0.85	− 0.76	− 0.25
Linguistic scale			0.83 (0.1)	0.87	0.66 − 0.91	−1.70	2.82
Linguistic comprehension			0.93 (0.07)	0.95	0.79 − 1.00	−1.73	3.58
Linguistic production			0.74 (0.12)	0.77	0.54 − 0.89	− 0.78	1.19
Extralinguistic scale			0.76 (0.09)	0.78	0.60 − 0.82	−1.87	3.69
Extralinguistic comprehension			0.86 (0.05)	0.85	0.79 − 0.93	0.02	− 0.38
Extralinguistic production			0.7 (0.19)	0.71	0.37 − 0.96	− 0.63	1.84
Paralinguistic scale			0.81 (0.09)	0.82	0.71 − 0.95	0.70	0.51
Paralinguistic comprehension			0.80 (0.06)	0.78	0.75 − 0.90	0.89	− 0.78
Paralinguistic production			0.82 (0.12)	0.79	0.67 − 1.00	0.42	−0.86
Context scale			0.72 (0.31)	0.84	0.21 − 0.96	−1.46	1.79
Context comprehension			0.78 (0.19)	0.82	0.43 − 0.93	−1.78	3.50
Context production			0.67 (0.38)	0.75	0.00–1.00	−1.27	1.53
Conversational scale			0.98 (0.03)	0.98	0.93 − 1.00	− 0.86	− 0.30

### Correlations with Education, Age and Gender

Table 3 shows the Pearson correlations between the ABaCo scales and subscales (plus the composite scores for comprehension and production and ABaCo global score) and education, age, and gender. The pattern of results mirrors that reported in the original norming sample (Angeleri et al., 2012) with the partial exception of Context comprehension scores, which in the original sample were not significantly correlated with age and showed a significant association with gender. As can be seen from Table 3, age consistently showed the largest correlations with performance, whereas gender was not significantly associated with the vast majority of the scores.

**Table 3:** Correlations of age, education and gender with ABaCo scales

	Age	Education	Gender (F)
Global score	− 0.50**	0.24**	0.02
Comprehension (all tasks)	− 0.45**	0.29**	0.00
Production (all tasks)	− 0.43**	0.13**	0.04
Linguistic scale	− 0.38**	0.20**	0.00
Linguistic comprehension	− 0.22**	0.15**	0.04
Linguistic production	− 0.38**	0.18**	− 0.03
Extralinguistic scale	− 0.39**	0.13**	0.21**
Extralinguistic comprehension	− 0.40**	0.28**	0.07
Extralinguistic production	− 0.29**	0.11*	0.13**
Paralinguistic scale	− 0.47**	0.22**	− 0.01
Paralinguistic comprehension	− 0.44**	0.25**	− 0.03
Paralinguistic production	− 0.31**	0.11*	0.01
Context scale	− 0.35**	0.10*	− 0.05
Context comprehension	− 0.24**	0.15**	− 0.05
Context production	− 0.31**	− 0.01	− 0.05
Conversational scale	0.09	0.07	− 0.03

* *p* <0.05; ** *p* <0.01

### Continuous Percentile Scoring

To allow a finer-grained approach to the scoring of ABaCo, we provide an Excel utility that takes as inputs the raw scores for the nine subscales plus the global score (range: 0–100), and converts them into percentile scores (see de Beurs et al., 2022) approximated to the nearest 5th percentile (range: 0–95). For example, a percentile score of 25 means that at least 25% (but less than 30%) of the reference population (i.e., adults with the same age and education as the patient) performs worse than the patient. A percentile score of 50 means at least 50% (but less than 55%) of the reference population performs worse than the patient; and a percentile score of 95 (the maximum score in the Excel utility) means that at least 95% of the reference population performs worse than the patient.

To convert raw score into percentile scores, we employ a quantile regression model (see Koenker & Hallock, 2001; Sherwood et al., 2016) with education, education squared, age, and age squared as predictors. The squared terms were included to account for curvilinear effects of age and education (e.g., performance first peaking and then declining with increasing age). This technique provides continuous estimates of the test score percentiles (e.g., the 50th percentile of the distribution of scores) conditional on one or more predictors. Because quantile regression employs all the information in the dataset to estimate the equations for the chosen percentiles, the resulting norms can be substantially more accurate than those derived by binning the data into age-by-education cells (as in Table 2), especially when some cells comprise a relatively small number of participants.

**Fig. 1:**
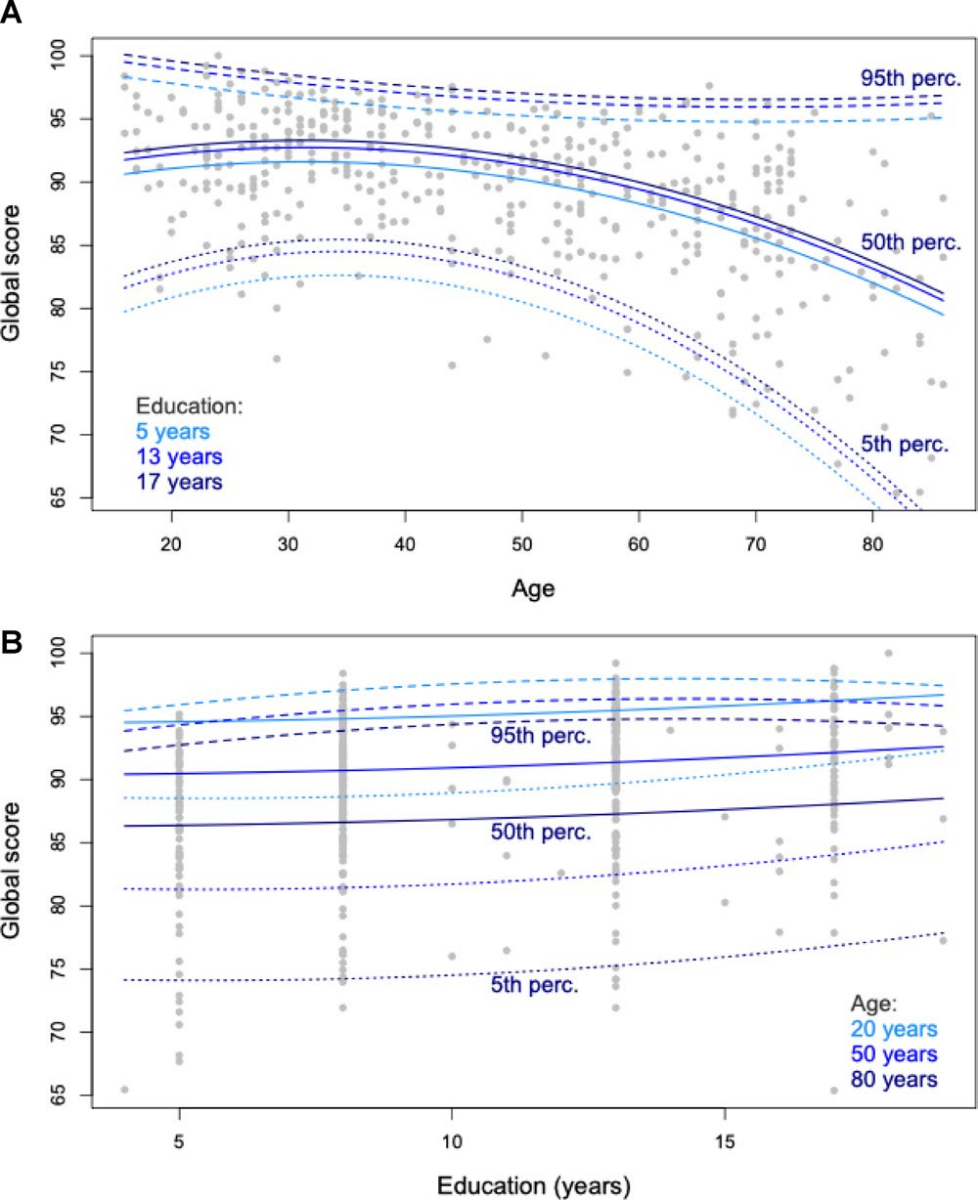
Illustration of the quantile regression estimates used to derive continuous percentile scores. **A** The 5th, 50th and 95th percentiles of the global score estimated as a function of age, for three levels of education (5, 13, and 17 years). **B** The 5th, 50th and 95th percentiles of the global score estimated as a function of education, for three levels of age (20, 50, and 80 years). In both panels, dotted lines indicate the 5th percentile, solid lines indicate the 50th percentile, and dashed lines indicate the 95th percentile. Note that, in order to develop the Excel utility described in the main text, quantile regression was used to estimate every 5th percentile from the 5th to the 95th (not shown here)

All analyses were performed in R 4.4.0 (R Core Team, 2024). For each ABaCo subscale plus the global score, we estimated the equations of every 5th percentile from the 5th to the 95th. Figure 1 illustrates the model results for the global score. In Fig. 1A, the 5th, 50th and 95th percentiles of the global score are estimated as a function of age, for three levels of education (namely 5, 13, and 17 years of education). In Fig. 1B, the same percentiles are estimated as a function of education, for three levels of age (namely 20, 50, and 80 years of age). The regression parameters are then used to calculate the nearest percentile score. The Excel utility to calculate percentiles from raw scores, together with the raw scores, the complete normative data and the R code used for the analysis, are available on OSF at the following link: https://osf.io/a9t2y/overview?view_only=325e6415699241dcab22639fa19889d9.

## Discussion

In this paper, we present an extension of the normative data for the ABaCo, a comprehensive tool for assessing various components of pragmatic ability. One of the main contributions of the present study is the inclusion of older adults in the normative sample, which now covers individuals from 15 to 86 years of age. This is particularly relevant in light of the demographic trends of population aging and the growing clinical need to evaluate pragmatic-communicative ability in older populations, who are at increased risk of both normative age-related decline and neurodegenerative conditions.

Pragmatic decline may be observed in healthy cognitive aging or serve as an early marker of pathological processes such as mild cognitive impairment or dementia (see Vegna et al., 2026). The extended norms presented here aim to enhance the accuracy and clinical utility of the ABaCo in aging populations, whose risk profiles are increasingly varied and complex. Recent studies, indeed, have demonstrated the existence of a range of pragmatic difficulties in healthy aging, involving not only language use and the discourse management (Petriglia, Marini et al., 2025; Hilviu et al., 2025; Marini et al., 2025) but also the gestures use, the integration of paralinguistic cues in speech, and the adherence to the norms of social appropriateness (e.g., Hilviu et al., 2022; Baraldi & Domaneschi, 2024; Bischetti et al., 2023). Moreover, longitudinal studies (e.g., Gabbatore et al. 2025a, 2026) have identified progressive patterns of decline in communicative-pragmatic ability with advancing age, particularly affecting comprehension skills. Such difficulties may lead to episodes of miscommunication and frustration which, in turns, can contribute to social isolation and be connected to a progressive increase of subjective cognitive decline (see Maffoni et al., 2022).

By including these older adults demographic group, the ABaCo norms become more representative and therefore more suitable for application in aging research and a wider range of clinical contexts. From a clinical perspective, indeed, the availability of extended norms has important implications. Accurate identification of pragmatic impairments allows for earlier and more precise diagnoses, facilitating objective evaluation of longitudinal changes, and enables assessment of the effectiveness of targeted training programs aimed at improving specific communicative weaknesses (see for example Bambini et al., 2020).

In recent years ABaCo has proven effective in evaluating changes in pragmatic performance before and after trainings programs involving people in neurological (Gabbatore et al., 2015; Parola et al., 2019), psychiatric conditions (Bosco et al., 2016), in the autism spectrum (Gabbatore et al., 2022), as well as healthy older adults (Gabbatore et al. 2026). The availability of updated and extended norms will further strengthen the reliability and interpretative power of these results in future research.

ABaCo represents one of the very few validated assessment tools for pragmatic ability, for which accurate and wide-ranging norms are available for the Italian population, together with the APACS (Arcara & Bambini, 2016). An additional strength of the present work is the inclusion of an Excel utility (based on quantile regression) that allows to easily convert raw scores into percentile scores, accounting for individual differences in age and education. This methodological improvement enhances the practical value of the instrument while also increasing the accuracy of performance interpretation.

## Data Availability

The Excel utility to calculate percentiles from raw scores, together with the raw scores, the complete normative data and the R code used for the analysis, are available on OSF at the following link: https://osf.io/a9t2y/overview?view_only=325e6415699241dcab22639fa19889d9.
